# Positive response to niraparib in chemo-refractory patients with metastatic appendiceal mucinous adenocarcinoma harboring ATM mutations: A case report

**DOI:** 10.3389/fonc.2023.1010871

**Published:** 2023-02-13

**Authors:** Junhui Wang, Huijuan He, Wansu Xu, Jianxin Chen

**Affiliations:** Department of Radiation Oncology, The Quzhou Affiliated Hospital of Wenzhou Medical University, Quzhou People′s Hospital, Quzhou, Zhejiang, China

**Keywords:** niraparib, chemo-refractory, appendiceal mucinous adenocarcinoma, ataxia-telangiectasia mutated (ATM), homologous recombination deficiency

## Abstract

**Background:**

Appendiceal mucinous adenocarcinoma, one kind of specific colorectal cancer, is lowly prevalent and rarely diagnosed in clinical practice. In addition, there have been limited standard treatment strategies established for patients with appendiceal mucinous adenocarcinoma, especially with metastatic disease. The regimens for colorectal cancer, which were adopted in appendiceal mucinous adenocarcinoma, usually resulted in limited effectiveness.

**Case presentation:**

Herein, we presented a case of chemo-refractory patient with metastatic appendiceal mucinous adenocarcinoma harboring ATM pathological mutation of exon 60, c.8734del, p.R2912Efs*26, and who has achieved a persistent response to salvage treatment of niraparib, with disease control time that reached 17 months and still in extension.

**Conclusions:**

We supposed that appendiceal mucinous adenocarcinoma patients harboring ATM pathological mutations may respond to the treatment of niraparib, even without a homologous recombination deficiency (HRD) status; however, it needs further confirmation in a larger cohort.

## Introduction

Appendiceal neoplasm is presented as one kind of rare tumor, which accounted for less than 1% of gastrointestinal tumors (1). Among those, appendiceal mucinous adenocarcinoma is even rare, which takes up approximately 0.2% to 0.3% of appendectomy specimens (2). Based on the rarity of the disease, the standard treatment strategy for appendiceal mucinous adenocarcinoma has not been well established (3). The mainstream treatment drugs for appendiceal mucinous adenocarcinoma have been based on the regimens established for colorectal cancer so far. However, the efficacy of anticancer agents including oxaliplatin, irinotecan, and 5-fluorouracil used in appendiceal mucinous adenocarcinoma cannot satisfy clinical demands because of the low response rate (3). It is an urgent need to investigate novel antitumor agents to promote the efficacy and hence prolong the survival time in such patients.

Ataxia-telangiectasia mutated (ATM) protein kinase, one of the master regulatory factors in the progress of DNA double-strand break response, is an important cell-cycle checkpoint kinase to maintain the stability of the genome (4, 5). The mutations or dysfunctions of the ATM gene have been detected during the carcinogenesis and development of various cancer types including breast cancer, ovarian cancer, and prostate cancer (6–8). In recent years, the efficacy-predicting role of ATM mutations for poly-ADP-ribose polymerase inhibitors (PARPi) in patients with ovarian cancer and metastatic castration-resistant prostate cancer has been preliminarily investigated (9, 10). Results of a prospective phase II trial implied that metastatic prostate cancer patients with ATM mutations, especially along with homologous recombination deficiency (HRD) ones, might be beneficial from the salvage treatment of PARPi (10). In addition, increasing clinical evidence has suggested that multiple kinds of cancer types including colorectal cancer, gastric cancer, and gallbladder cancer harboring ATM mutations may profit from PARPi treatment (11–13). Herein, we presented a case of chemo-refractory metastatic appendiceal mucinous adenocarcinoma, whose results of the gene test suggested ATM pathological mutations, has achieved a persistent response to the palliative treatment of niraparib, and hence resulted in progression-free survival (PFS) time that reached 17 months and still in extension.

## Case presentation

**Figure 1:**
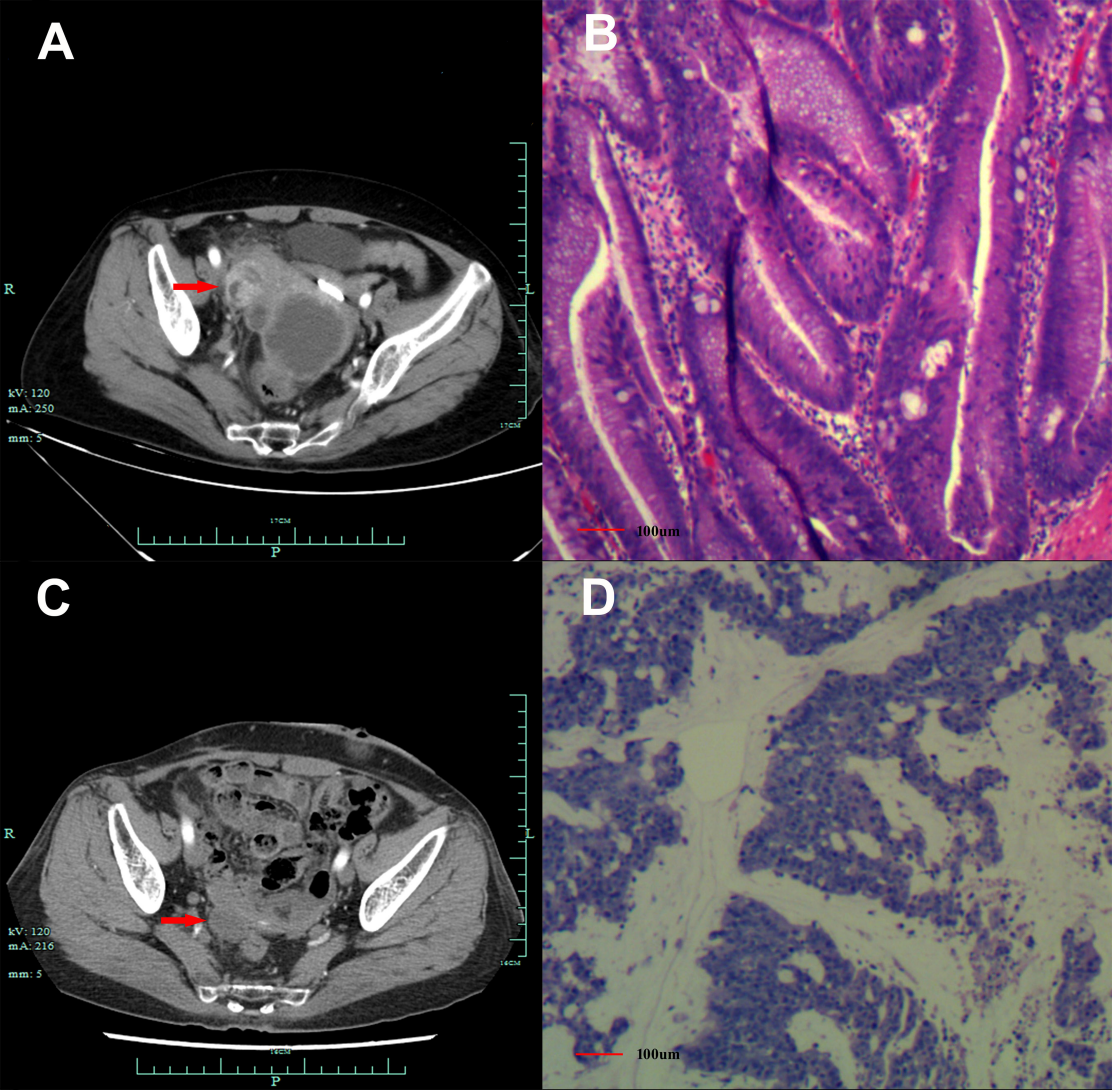
Abdomen CT scans and histological findings. **(A)** Abdomen CT scans suggested emerging mass in the vermiform appendix (red arrow), surrounded by multiple abscesses on 26/08/2019. **(B)** Histological finding with hematoxylin and eosin staining from the first surgery in September 2019. **(C)**. Abdomen CT scans suggested an emerging occupation behind the uterus (red arrow), along with multiple nodes on the omentum suggesting recurrence on 10/07/2020. **(D)** Histological finding with the hematoxylin and eosin-stained biopsy specimen from the second cytoreductive surgery on 20/07/2020.

**Figure 2:**
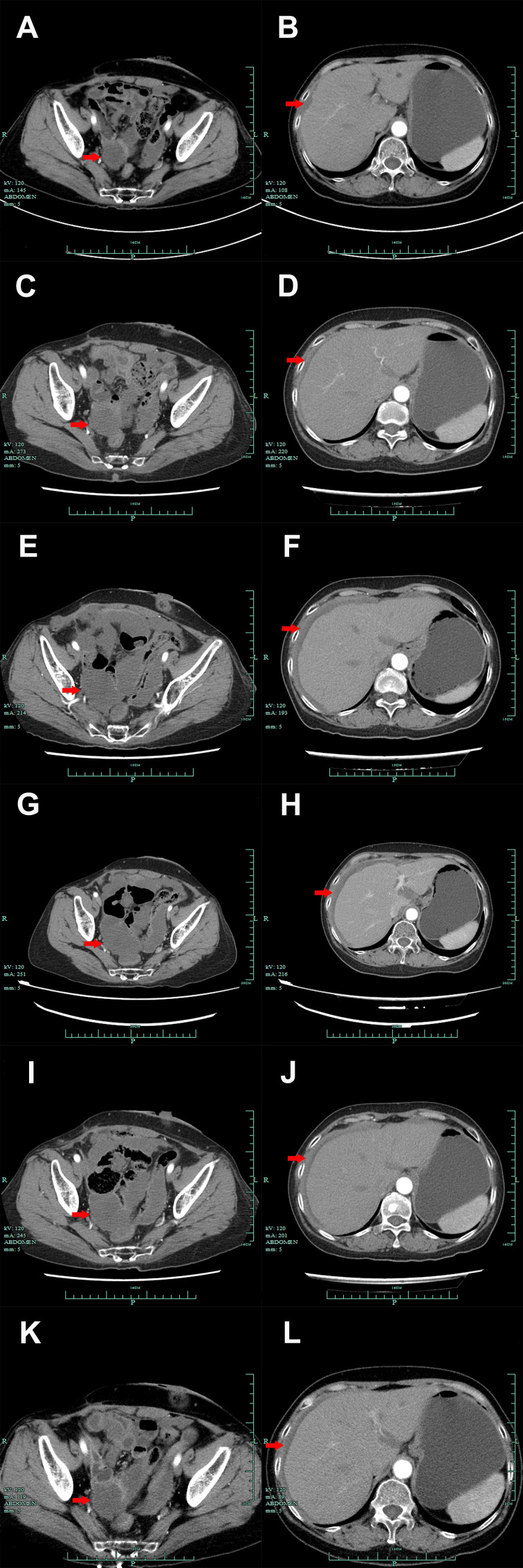
Abdomen CT scan presentations suggested the emerging occupation behind the uterus (**A, C, E, G, I, K**, red arrow) and thickened peritoneum (**B, D, F, H, J, L**, red arrow) from October 2020 to March 2022. **(A, B)**. October 2020. **(C, D)**. December 2020. **(E, F)**. January 2021. **(G, H)**. May 2021. **(I, J)**. September 2021. **(K, L)**. March 2022.

**Figure 3:**
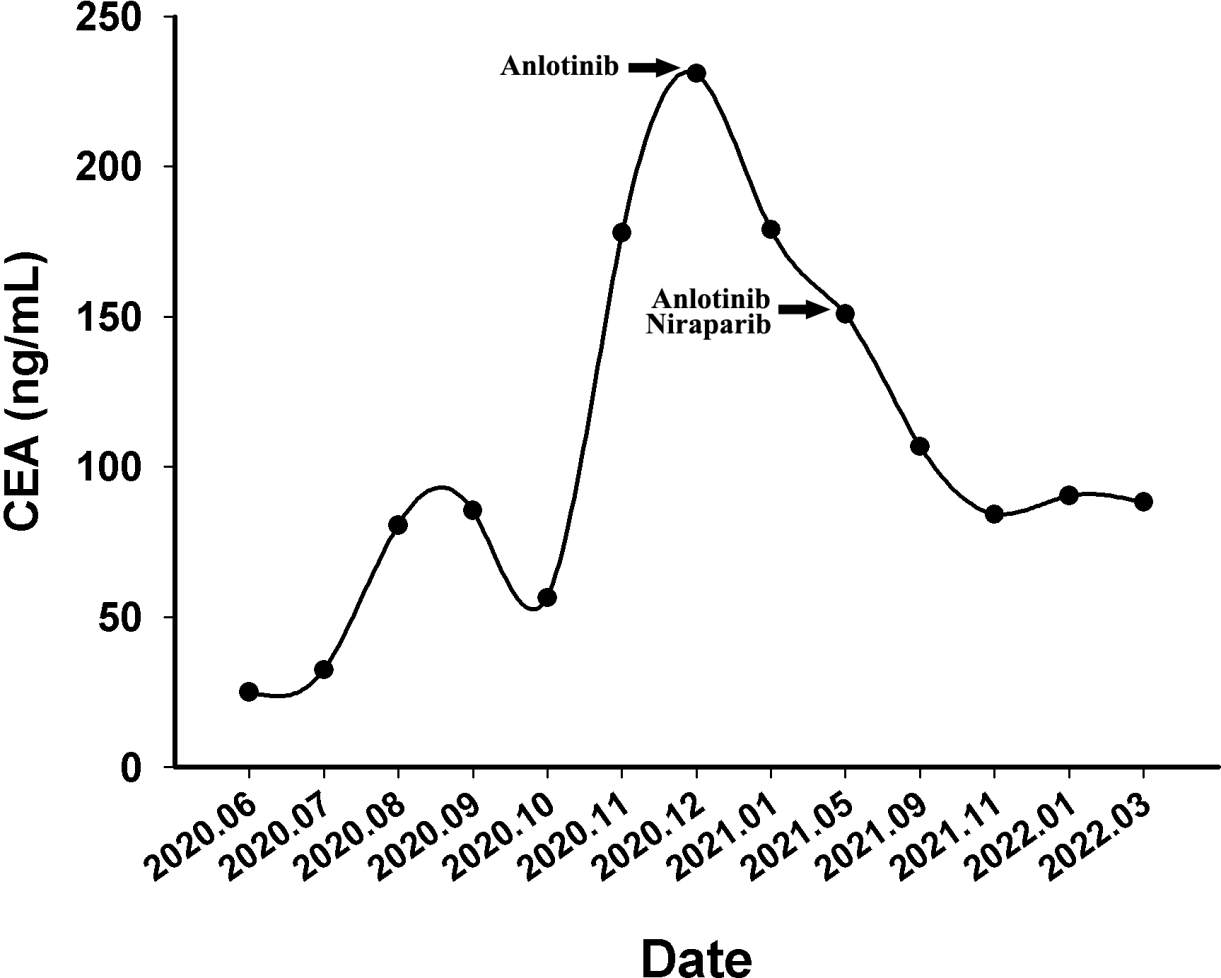
The variations of tumor marker CEA (normal range 0 to 5 ng/mL) from June 2020 to March 2022.

A 60-year-old Chinese woman was admitted to our hospital on 26/08/2019 with colporrhagia for 2 days. In addition, the patient denied smoking, alcohol, or any other medical or family history. Pelvic CT showed an emerging mass in the vermiform appendix, surrounded by multiple abscesses (**Figure 1A**). In addition, there was no occupation detected in the liver, lungs, or abdomen. Subsequently, the patient received radical appendectomy for tumors, histological findings of which suggested appendiceal mucinous adenocarcinoma, without invasion to the surrounding nerves or vessels (**Figure 1B**). Immunohistochemistry findings were presented as the following: CDX2 (positive), CK7 (negative), CK (positive), CK20 (positive), CEA (positive), ER (negative), PR (negative), CA125 (negative), WT-1 (negative), and Ki-67 (60%). Based on those, a diagnosis of appendiceal mucinous adenocarcinoma was made and staged as pT3N0M0 according to the American Joint Committee on Cancer (AJCC) version 8th. Subsequent therapy with the regimen of CapeOX (oxaliplatin 130 mg/m^2^ on day 1 and oral capecitabine 1,000 mg/m^2^ twice a day, from days 1 to 14, every 21 days) has been scheduled as adjuvant treatment. However, the patient was admitted to our hospital again for colporrhagia and abdominal pain in July 2020, 4 months after the completion of adjuvant therapy. Repeated abdomen CT revealed an emerging occupation behind the uterus, along with multiple nodes on the omentum, suggesting recurrence (**Figure 1C**). Palliative cytoreductive surgery was performed to remove the major lesion behind the uterus on 20/07/2020. Histological findings implied recurrence of appendiceal mucinous adenocarcinoma (**Figure 1D**). In terms of the existence of multiple lesions on the omentum, a systematic regimen of FOLFIRI (irinotecan 180 mg/m^2^, on day 1, leucovorin 400 mg/m^2^, 5-fluorouracil 400 mg/m^2^ intravenous injection followed by 5-fluorouracil 2,400 mg/m^2^ pump injection for 46 h, every 14 days) was prescribed as palliative treatment. After three cycles’ exposure, emerging occupation behind the uterus (**Figure 2A**) as well as the emergence of thickened peritoneum (**Figure 2B**) was presented by repeated abdomen CT scans, which suggested progressive disease (PD). As salvage therapy, a subsequent regimen of BOL (bevacizumab 5 mg/kg on day 1, oxaliplatin 85 mg/m^2^ on day 1, and Letitrexed 3 mg/m^2^ on day 1, every 14 days) was arranged from October to December 2020. However, efficacy evaluation by regular CT scans still resulted in PD again (**Figures 2C, D**). Beyond that, subsequent monotherapy with anlotinib, a multiple-target agent, failed to control the disease either (**Figures 2E, F**). The characteristic of the recurrent appendiceal mucinous adenocarcinoma seems chemo-refractory. In order to screen potentially available targets, a whole-exome sequencing (WES) with next-generation sequencing (NGS) using tissue and plasma sample was performed. Results of the NGS revealed ATM exon 4 p.N81Tfs*20 by frequency as 11.59%, as well as ATM exon 60 p.R2912Efs*26 by frequency as 10.46%, and KRAS exon 2 p.G13D by frequency as 17.94%. However, homologous recombination deficiency (HRD) was not detected with the results of the NGS analysis. Based on the established efficacy of poly ADP-ribose polymerase inhibitor (PARPi) in castration-resistant prostate cancer harboring ATM mutations (10), a combination treatment of niraparib and anlotinib was administrated as salvage regimen. After 3 months’ exposure, the target lesion as well as the multiple nodes was suggested as stable disease (reduction by 15.8%, **Figures 2G, H**) according to the CT scans in May 2021. In addition, tumor markers of CEA (normal range, 0 to 5 ng/ml) decreased significantly during the combination treatment (**Figure 3**). The combinational regimen, hence, has been continued as salvage treatment from then on. Regular abdomen CT scans suggested persistent stable disease for efficacy evaluation (**Figures 2I–L**). The patient still receives the combinational regimen (niraparib at a dose of 200 mg Qd and anlotinib at a dose of 10 mg Qd, day 1 to day 14, every 21 days) as maintenance treatment now. There was no treatment-related adverse event observed during the combinational treatment. The variation of CEA during the whole treatment is presented in the **Figure 3**. The variation of tumor size for target lesion is shown in **Supplementary Table 1**.

## Discussion

We herein presented a case of chemo-refractory metastatic appendiceal mucinous adenocarcinoma, whose results of the gene test suggested ATM pathological mutations, has achieved persistent response to the palliative treatment of niraparib, and hence resulted in PFS time that reached 17 months and still in extension.

The ATM gene emerged as a significant regulator included in DNA damage repair (DDR) mechanisms, which is responsible for the repair of double-strand breaks. The potential mechanisms for the repair function of ATM are induction of cell-cycle arrest *via* TP53 and initiation of the repair process of DNA damage by the activation of BRCA1/2 (11). Dysfunction of related proteins caused by the mutation of the ATM gene may lead to the occurrence of various cancer types including ovarian cancer, prostate cancer, pancreatic cancer, colorectal cancer, and breast cancer (14). In cancer patients harboring ATM mutations or dysfunction, the clinical application of PARPi may be possible to cause the effect of lethal synthesis and result in favorable antitumor efficacy. TOPARP-B was a phase II prospective clinical trial conducted to validate the association between DDR gene aberrations (BRCA1/2 mutations as 33%, ATM mutations 21%, CDK12 mutations as 21%) and response to olaparib, one of PARP inhibitors, in metastatic castration-resistant prostate cancer. Patients with DDR gene aberrations were randomly assigned (1:1) to receive oral olaparib 300 mg twice daily or placebo. The results presented that the median PFS was 13.8 months (95% CI, 10.8 to 20.4) with olaparib and 8.2 months (95% CI, 5.5–9.7) with placebo (hazard ratio, 0.65, 95% CI, 0.44 to 0.97, *P* = 0.034). The objective response for the subgroup of ATM aberrations was observed in 7/19 patients, ranking only second to BRCA1/2 (25/30) and PALB2 (4/7) (10). Results of the study suggested that there existed a subset of patients with ATM-altered metastatic castration­resistant prostate cancer that appeared to derive benefit from olaparib (13). In addition, there were preclinical studies reporting that PARPi might also be effective in ATM-deficient cancers including colorectal cancer (15, 16). Regarding the patient in the present case, appendiceal mucinous adenocarcinoma cells appeared to be refractory to cytotoxic drugs including oxaliplatin, irinotecan, and 5-fluorouracil, which are a recommended option as treatment in colorectal cancer. We considered that ATM aberrations may contribute to the drug resistance, which was consistent to former literature (17). In such circumstance in the present case, it was extremely difficult to select potential effective strategies as further line treatment. Based on the NGS outcomes, salvage treatment of niraparib was finally attempted because of the ATM mutations and unexpectedly resulted in satisfactory effectiveness.

Although no prospective studies have been conducted to investigate the efficacy of PARPi in colorectal cancer patients harboring ATM mutations, there are limited case studies to report that. Most recently, there has been a case report on a pretreated patient diagnosed with stage IV ATM-deficient colorectal cancer, who was effectively treated with an olaparib–irinotecan combination after exhaustion of all available treatment choices (11). The duration of response for the PARPi patient in that case lasted for 4 months, but finally resulted in progression (11). The alteration sites of ATM in that patient were NM_000051.3:c.8925_8928dup: p.(Glu2977Argfs*2) and NM_000051:c.3880dup: p.(Ile1294Asnfs*8), which were different from the mutations in the present case (exon 4 c.238_241dup, p.N81Tfs*20 by frequency as 11.59%, as well as exon 60, c.8734del, p.R2912Efs*26 by frequency as 10.46%). After the searching in *GeneCards*, the mutation of exon 60, c.8734del, p.R2912Efs*26 may have emerged as a pathological mutation, which might contribute to the effectiveness of PARPi in the present case. However, a direct testing on organoids or patient-derived xenografts might be more useful to identify the determinate responsible mutation.

The leading limitation of the present case report comes from the nature of a case report. Although we supposed that the effect of disease control resulted from the introduction of niraparib, the effectiveness of niraparib in appendiceal mucinous adenocarcinoma harboring ATM mutations still needs further identification in basic or vivo experiments, and even in clinical research.

Briefly, we presented a case of chemo-refractory patient with metastatic appendiceal mucinous adenocarcinoma harboring ATM mutations and who has achieved a persistent response to salvage treatment of niraparib, with disease control time that reached 17 months and still in extension. We also suggested that NGS analysis might be helpful occasionally, for patients of treatment-refractory cancer such as metastatic appendiceal mucinous adenocarcinoma in the present case.

## Data availability statement

The original contributions presented in the study are included in the article/**Supplementary Material**. Further inquiries can be directed to the corresponding author.

## Ethics statement

The studies involving human participants were reviewed and approved by Ethical Committee of People′s Hospital of Quzhou. The patients/participants provided their written informed consent to participate in this study.

## Author contributions

JW: Conceptualization, Methodology, Software, Writing- Original draft preparation, Software, Validation. HH: Data curation, Supervision. WX: Visualization, Investigation. JC: Data curation, Supervision, Writing-Reviewing and Editing. All authors contributed to the article and approved the submitted version.
