# Supplementary material for: Positive response to niraparib in chemo-refractory patients with metastatic appendiceal mucinous adenocarcinoma harboring ATM mutations: A case report
Source: Front Oncol. 2023 Feb 13;13:1010871. doi: 10.3389/fonc.2023.1010871 (PMC9969135; doi:10.3389/fonc.2023.1010871)
Supplement: Supplementary file 1 [file Table_1.docx]

**Table 1**. Varied size of the targeted lesion in pelvic cavity during the whole treatment according to RECIST version 1.1.

| **Date** | **Exposure** | **Duration** | **Targeted lesion in pelvic cavity**  **(LD×SD, millimeter)** |
| --- | --- | --- | --- |
| 2020.06 | Recurrence |  | 21×16 |
| 2020.10 | FOLFIRI | 4 months | 38×27 |
| 2020.12 | BOL | 2 months | 51×34 |
| 2021.01 | Anlotinib | 1.5 months | 57×33 |
| 2021.05 | Anlotinib, Niraparib |  | 48×31 |
| 2021.09 | Anlotinib, Niraparib |  | 42×29 |
| 2022.03 | Anlotinib, Niraparib | 14 months | 41×29 |

**Abbreviations**: LD, Longest Diameter; SD, Shortest Diameter; FOLFIRI, Irinotecan + Fluorouracil + Leucovorin; BOL, Bevacizumab + Oxaliplatin + Letitrexed.
